# Supplementary material for: Malaria parasite DNA-harbouring vesicles activate cytosolic immune sensors
Source: Nat Commun. 2017 Dec 7;8:1985. doi: 10.1038/s41467-017-02083-1 (PMC5719353; doi:10.1038/s41467-017-02083-1)

**Supplementary Figure 1**

**Supplementary
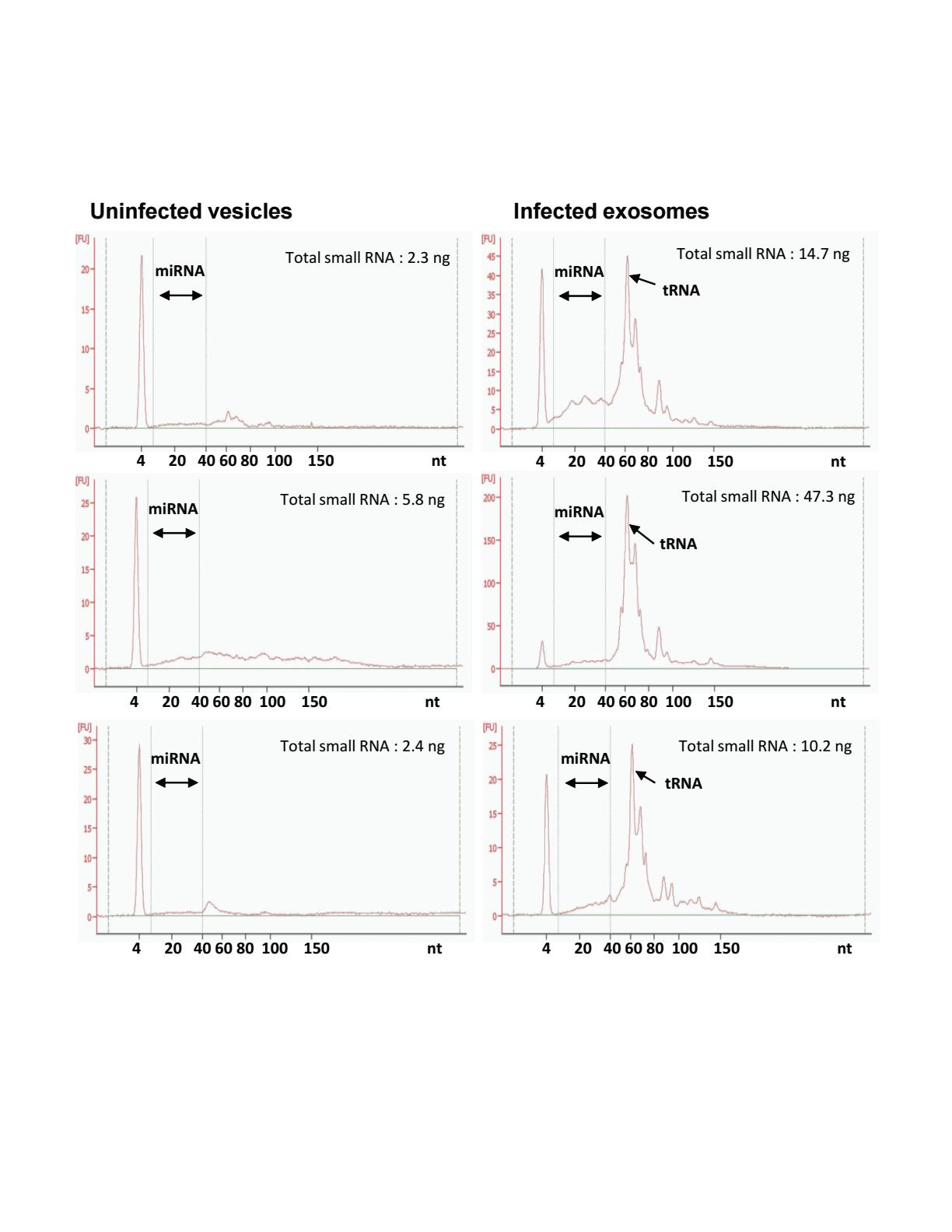
Figure 1.** EVs from iRBCs contain more RNA species than do uRBC vesicles. RNA was extracted from uRBC vesicles (n = 3) and EVs from iRBCs (n = 3). The small RNA profile was assessed using an Agilent^®^ 2100 Bioanalyzer™ instrument with the Agilent^®^ Small RNA Kit, which provides the yield of small RNAs between 4 nt – 200 nt.

**Supplementary Figure S2**

**
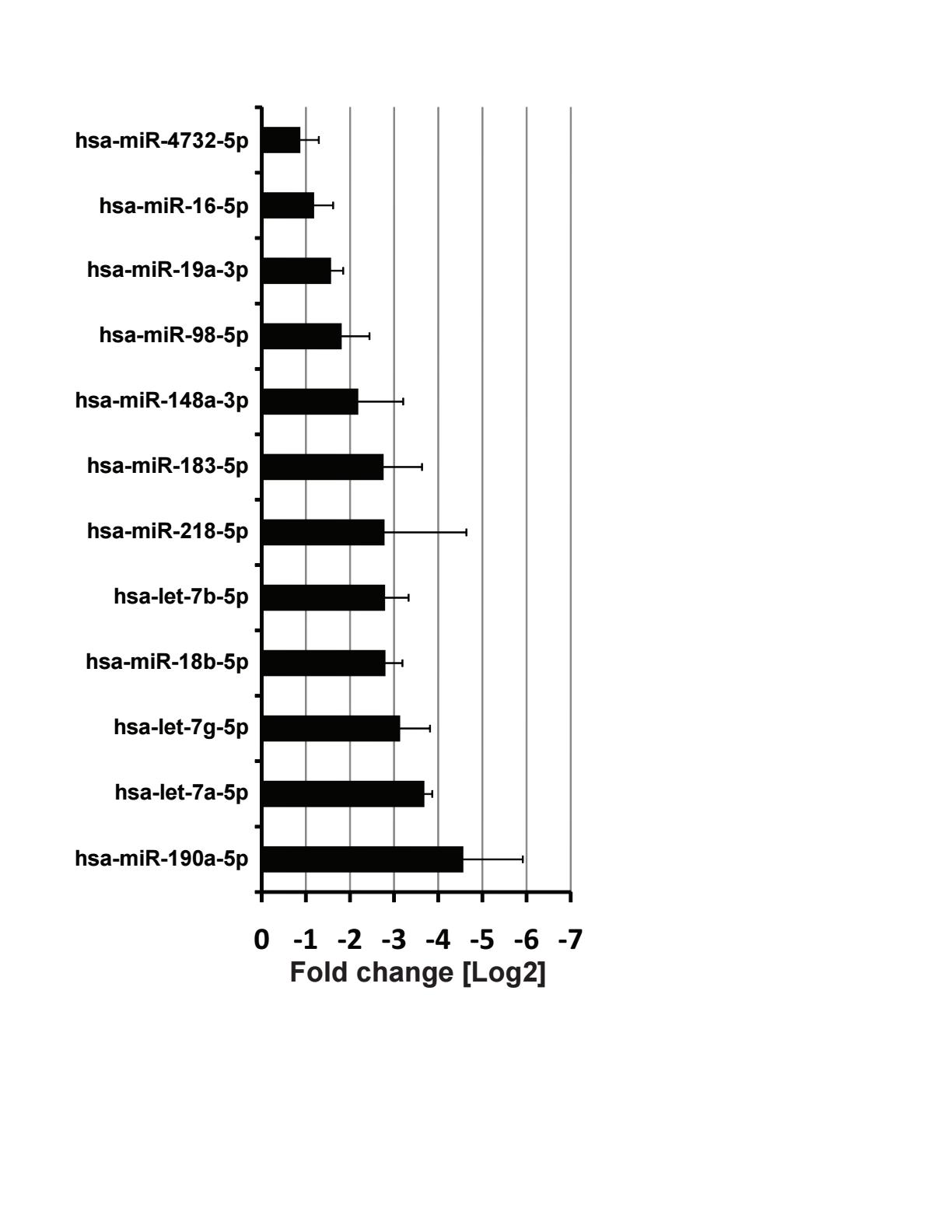
**

**Supplementary Figure 2**. Validation of differently expressed human miRNAs in EVs from iRBCs by qRT-PCR. Human miRNAs were differentially expressed (p (inf-ex vs uninf-v) ≤ 0.05 and ± 2.0 fold change) in EVs from iRBCs, and vesicles from uRBCs in the NGS study were further validated using qRT-PCR. In addition, miRNAs with sequencing reads greater than 20 were validated which is the level of detection of qRT-PCR. The Delta delta Ct (ΔΔCt) method was used to calculate fold change using hsa-miR-19b as a normalisation control, which was found to be the most consistently expressed protein across all vesicle samples.


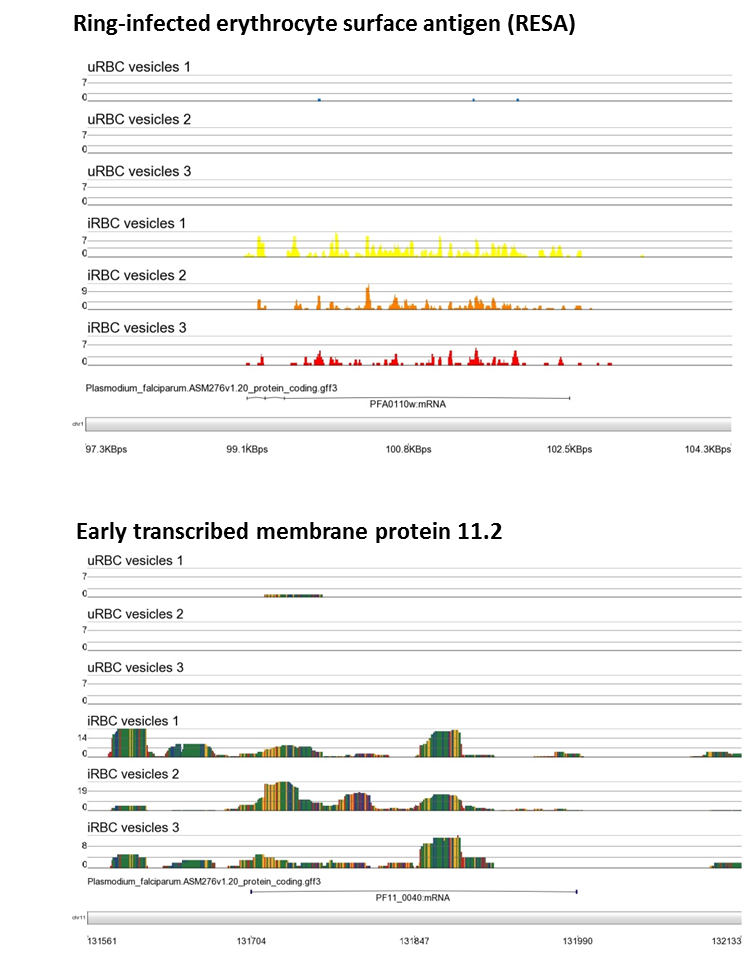


**Supplementary Figure 3**

**Supplementary Figure 3.** Deep sequencing coverage of *P. falciparum* ETMP11.2 coding mRNA detected in EVs secreted from iRBCs. Reads aligned to ASM276v1 were mapped to EnsemblProtist non-coding RNA annotations using the Partek GS miRNA seq pipeline. Remaining reads were then passed through the Partek GS mRNA seq pipeline and mapped to EnsemblProtists protein coding annotations. Overlapping regions were merged and visualized using a Partek GS genome browser. This figure displays the sequencing reads identified as covering the chromosome location of ETMP11.2.

**Supplementary Figure 4**


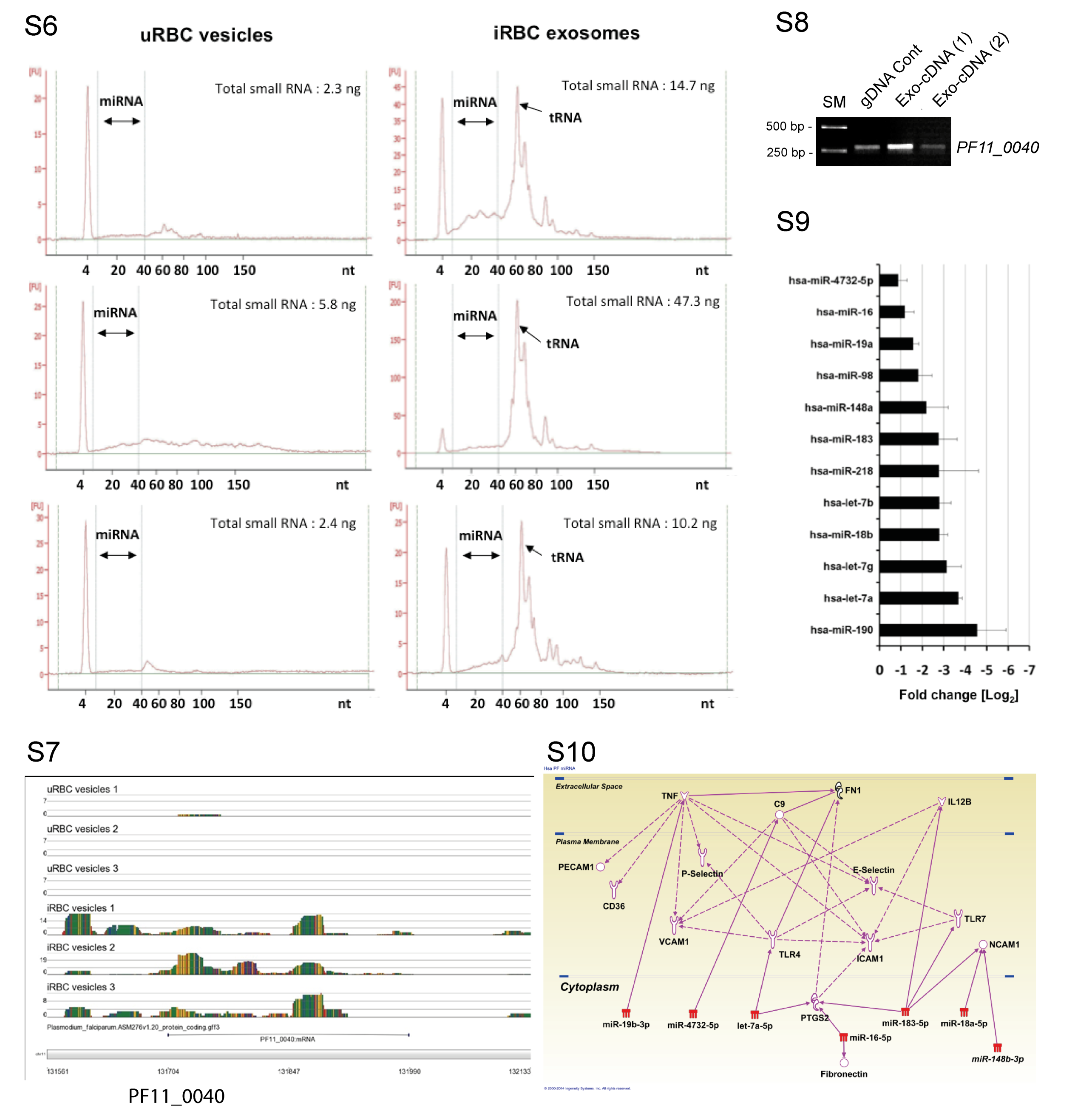


Ev-cDNA (1)

Ev-cDNA (2)

**Supplementary Figure 4.** EVs were purified (two independent biological repeats, samples 1 and 2) and subjected to RNA extraction. The cDNA reaction was performed on the RNA elute following PCR amplification using primers for the full-length PF11_0040 gene. PCR amplification using parasite gDNA served as a length control. Cont- control.

**Supplementary Figure 5**


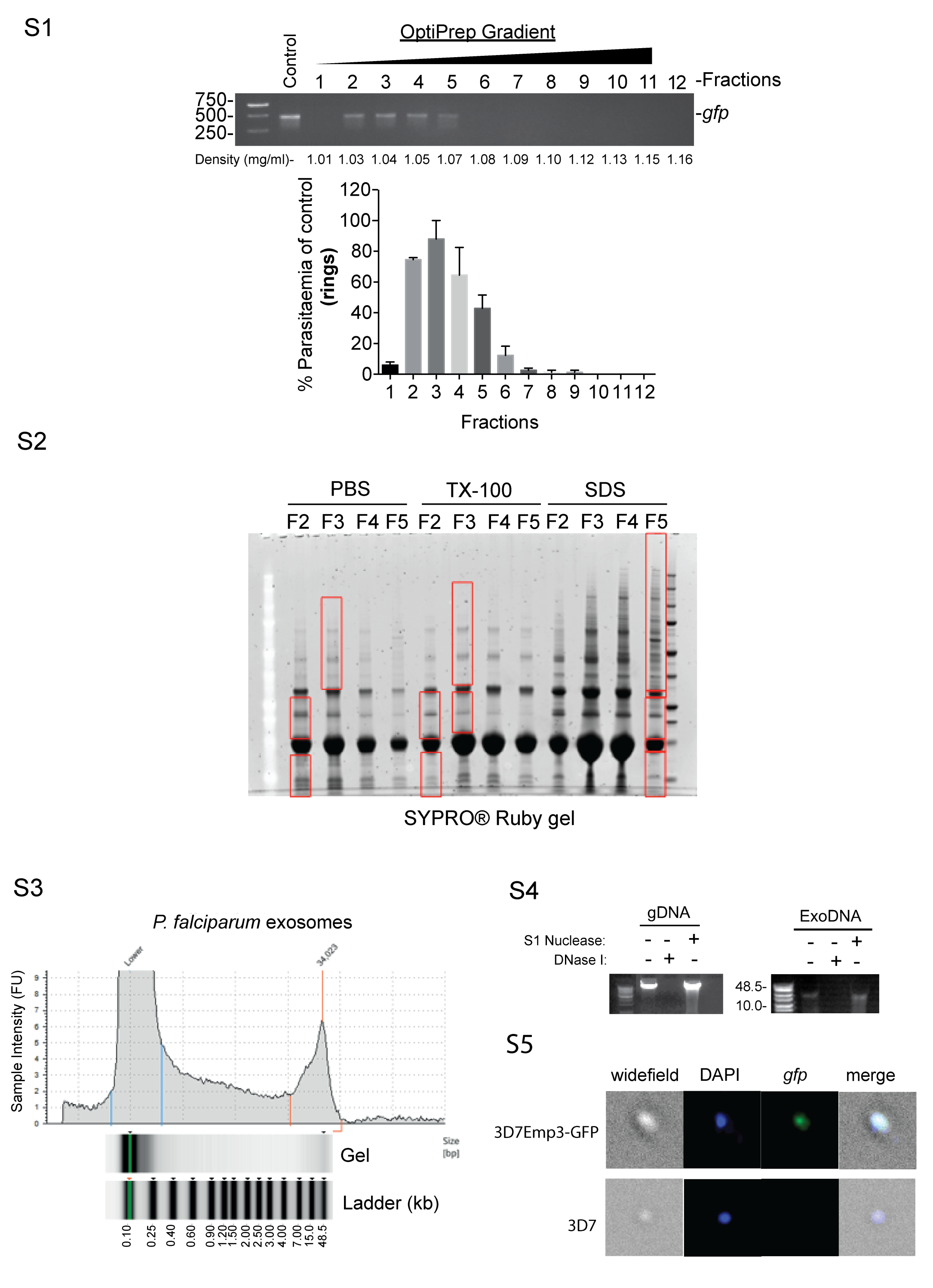


***P. falciparum* vesicles**

**Supplementary Figure 5.** The presence of gDNA from a mixed culture of 3D7 and CS2 ring-stage *P. falciparum*-derived EVs was confirmed using an Agilent 2200 TapeStation detection kit.

**Supplementary Figure 6**


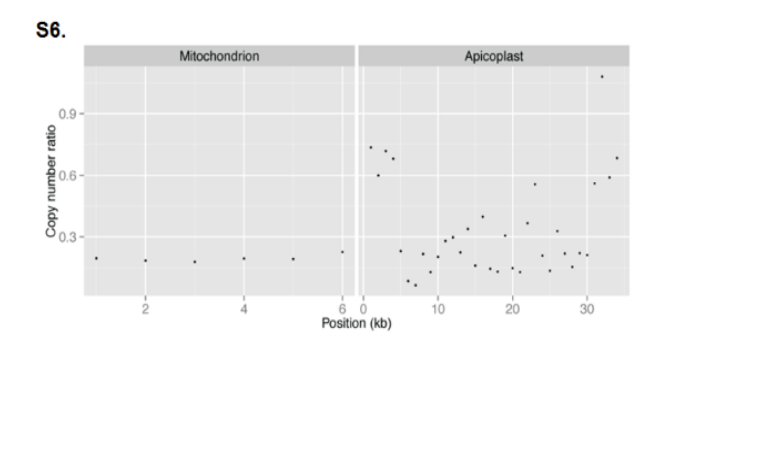


**Supplementary Figure 6.** RCNFC for mitochondrial and apicoplast chromosomes. Eight copies of the mitochondrial genome are present in the EVs, compared with 45 in the control. Apicoplast coverage is variable (mean fold change 0.2).


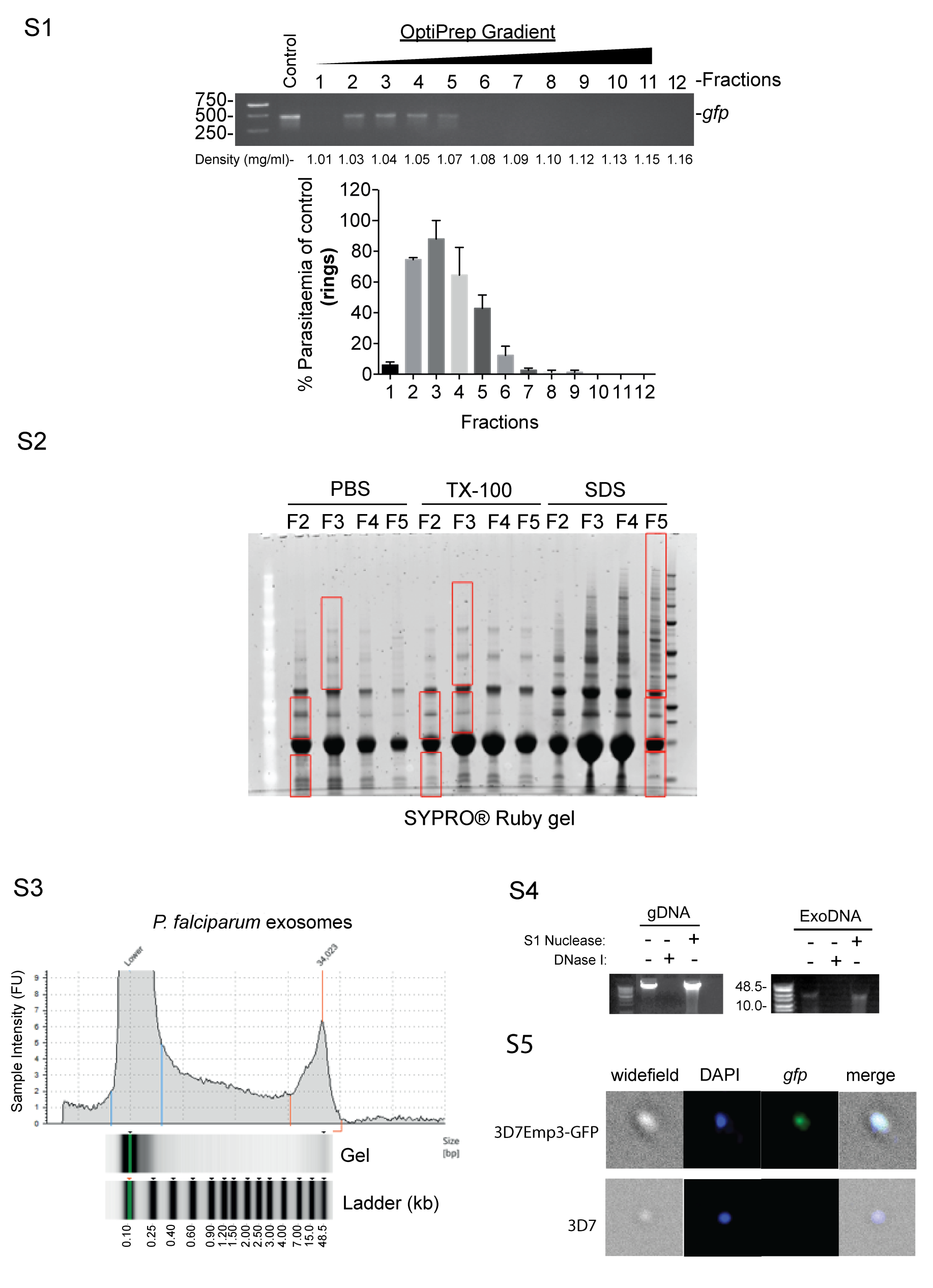


**Supplementary Figure 7**

**Supplementary Figure 7.** The Ev-FISH images for the gfp plasmid of EVs from ring-stage iRBCs. The gfp signal is detected in EVs from a cell line transfected with the plasmid (3D7Emp3-GFP) but not in EVs from a 3D7 wild type cell line. In both cases EVs carry DNA, as shown by the DAPI signal.

**Supplementary Figure 8**


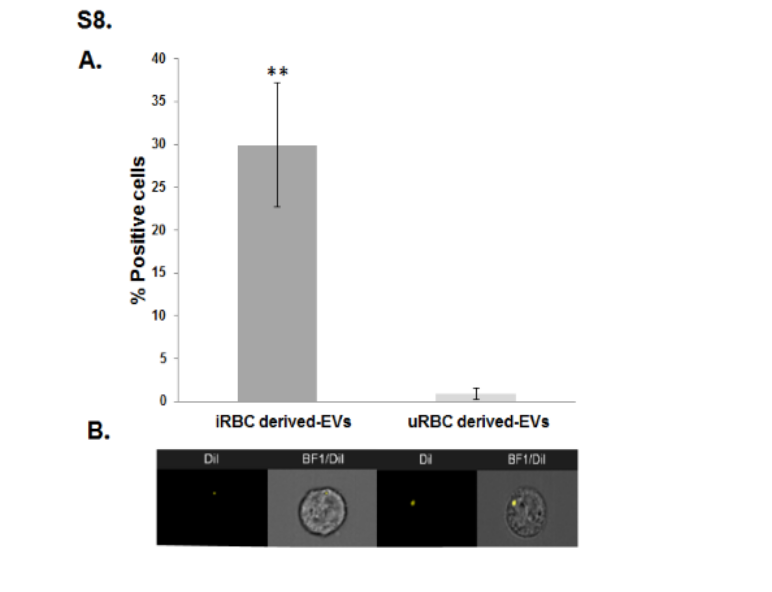


**B.**

**A.**

**Supplementary Figure 8**. Internalization (uptake) of *Pf*-derived EVs into monocytes as visualized by IFC. **A**. EVs were stained with lipid dye Dil, introduced into monocytes (THP-1 cells) for 5 minutes and then vesicle uptake was imaged using IFC, n=3, T-test analysis **(p≤0.001). **B**. EVs are detected as spots inside the recipient cells; BF- bright field.

**Supplementary Figure 9**


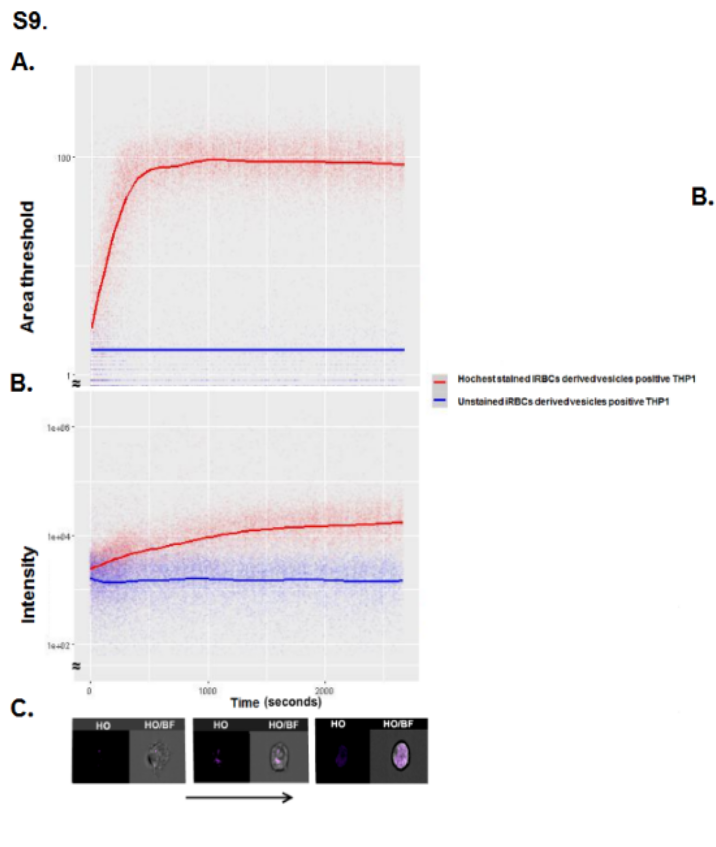


**Supplementary Figure 9.** Kinetic measurement of *Pf* EV uptake by monocytes using IFC. *Pf-* derived EVs were labeled by HO and the uptake into THP-1 cells was measured over 45 minutes.

**A**. The graph represents the area of the highest intensity stained pixels (Area_Threshold) of the HO staining with time. Small bright spots have a low area, and as the hoechst evenly distributes throughout the cell, the area increases. **B**. The graph represents HO intensity detected over time originating from monocyte recipient cells, demonstrating HO accumulation in the cells. **C.** Images of three representative recipient cells, from different time points. Within the first 15min, the EV Hoechst signal appears as clear bright spots inside the cells, followed by signal dispersal throughout the cell. This is demonstrated by the increase in the area of the highest intensity pixels (Area_Threshold) depicted in (A).

**Supplementary Figure 10**


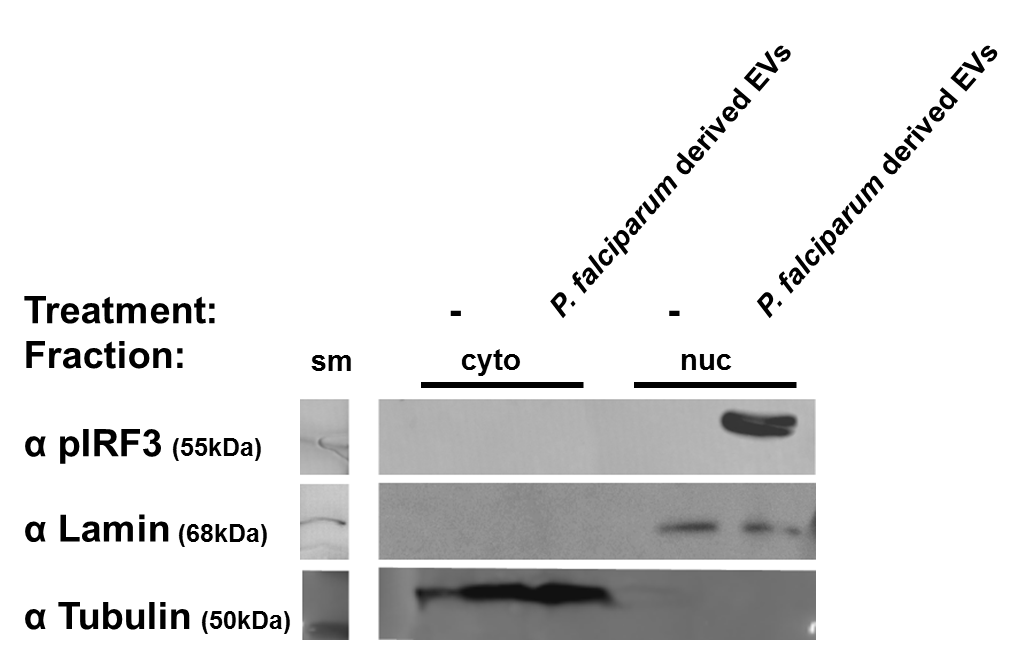


**Supplementary Figure 10.** Subcellular fractionation (cytosol and nucleus compartments). THP-1 cells were incubated with *Pf* ring stage-derived vesicles for 24h; cells were then fractionated for two fractions of the cytoplasm and nuclear. WB analysis was performed for pIRF3, Tubulin (as a cytosolic control) and Lamin (as a nuclear control). Cyto- cytosolic fraction, nuc- nuclear fraction. sm-size marker.

**Supplementary Table 1. Percentage of alignment and mapping of RNA to *H.Sapiens* and *P.falciparum* genome and annotations in exosomes secreted from *P.falciparum*-infected RBCs and uninfected vesicles**.

|  | **Uninf-v** | | **Inf-ex** | |
| --- | --- | --- | --- | --- |
|  | **Reads** | **%** | **Reads** | **%** |
| ***H.Sapien aligned*** | *1852864* | *83.70* | *1737494* | *57.18* |
| hsa_miRNA | 692646 | 31.29 | 293578 | 9.66 |
| hsa_snordRNA | 20 | 0.00 | 188 | 0.01 |
| hsa_snoRNA | 72 | 0.00 | 86 | 0.00 |
| hsa_yRNA | 1232 | 0.06 | 4340 | 0.14 |
| hsa_tRNA | 9053 | 0.41 | 35477 | 1.17 |
| hsa_lincRNA | 498 | 0.02 | 1725 | 0.06 |
| hsa_rRNA | 1342 | 0.06 | 951 | 0.03 |
| hsa_coding RNA | 76705 | 3.47 | 103136 | 3.39 |
| hsa_unannotated RNA | 1071295 | 48.40 | 1298013 | 42.72 |
|  |  |  |  |  |
| ***P. falciparum aligned*** | *7141* | *0.32* | *350807* | *11.54* |
| P.fal_tRNA | 137 | 0.01 | 9814 | 0.32 |
| P.fal_coding RNA | 120 | 0.01 | 6962 | 0.23 |
| P.fal_non_coding RNA | 55 | 0.00 | 5711 | 0.19 |
| P.fal_unannotated RNA | 6829 | 0.31 | 328320 | 10.80 |
|  |  |  |  |  |
| Unaligned | 353752 | 15.98 | 960266 | 31.60 |
| **Total raw reads** | **2213620** |  | **3038753** |  |

**Supplementary Table 2. Plasmodium Falciparum non-coding RNA contained in iRBC exosomes**

| **Chromosome** | **Start** | **Stop** | **Strand** | ***P.fal* ncRNA** | **Raw reads** |
| --- | --- | --- | --- | --- | --- |
| 13 | 2797363 | 2797840 | + | PF13TR011:ncRNA | 3963 |
| 8 | 92521 | 98696 | - | MAL8b_28s:ncRNA | 661 |
| 8 | 100014 | 100176 | - | PF08_tmp2:ncRNA | 279 |
| 12 | 576430 | 576565 | + | PF12TR006:ncRNA | 147 |
| 13 | 2799275 | 2799446 | + | RNAzID:1891:ncRNA | 135 |
| 14 | 1889356 | 1889473 | + | U5RNA:mRNA | 112 |
| 14 | 981211 | 982552 | + | PF14TR004:ncRNA | 72 |
| 3 | 398132 | 398251 | - | RNAzID:2846:ncRNA | 58 |
| 12 | 1857841 | 1858075 | + | PF12TR015:ncRNA | 53 |
| 14 | 782332 | 782399 | - | RNAzID:2110:ncRNA | 46 |
| 13 | 243882 | 244061 | - | RNAzID:1537:ncRNA | 42 |
| 5 | 465774 | 465842 | - | RNAzID:3370:ncRNA | 21 |
| 14 | 979397 | 979587 | + | RNAzID:2132:ncRNA | 17 |
| 13 | 1213708 | 1213908 | + | RNAzID:1678:ncRNA | 15 |
| 12 | 1857766 | 1858023 | + | RNAzID:1335:ncRNA | 13 |
| 9 | 762972 | 763081 | + | RNAzID:4653:ncRNA | 5 |
| 10 | 1010599 | 1013746 | + | PF10TR008:ncRNA | 4 |
| 12 | 775387 | 780094 | + | PF12TR008:ncRNA | 3 |
| 12 | 1734910 | 1743841 | + | PF12TR014:ncRNA | 3 |
| 14 | 2215 | 5393 | + | PF14TR001:ncRNA | 3 |
| 4 | 595385 | 595542 | + | RNAzID:3145:ncRNA | 3 |
| 7 | 1409078 | 1410935 | + | PF07TR005:ncRNA | 3 |
| 9 | 324625 | 327466 | + | PF09TR001:ncRNA | 3 |
| 7 | 106489 | 108925 | + | PF07TR002:ncRNA | 2 |
| 1 | 219186 | 219462 | + | PF01TR001:ncRNA | 2 |
| 1 | 310558 | 311428 | + | PF01TR002:ncRNA | 2 |
| 11 | 512569 | 516136 | + | PF11TR002:ncRNA | 2 |
| 12 | 161842 | 161904 | + | RNAzID:1075:ncRNA | 2 |
| 12 | 575533 | 576286 | + | PF12TR005:ncRNA | 2 |
| 13 | 1676588 | 1676678 | - | RNAzID:1750:ncRNA | 2 |
| 14 | 982830 | 982890 | - | RNAzID:2136:ncRNA | 2 |
| 14 | 3249455 | 3250499 | + | PF14TR010:ncRNA | 2 |
| 5 | 956923 | 957565 | + | PF05TR004:ncRNA | 2 |
| 8 | 1276696 | 1279867 | + | PF08TR005:ncRNA | 2 |
| 5 | 1288552 | 1289302 | + | PF05TR005:ncRNA | 1 |
| 8 | 240995 | 241057 | - | RNAzID:4268:ncRNA | 1 |
| 12 | 1745293 | 1745458 | + | RNAzID:1320:ncRNA | 1 |
| 4 | 580160 | 580317 | + | RNAzID:3134:ncRNA | 1 |
| 10 | 411613 | 412448 | + | PF10TR004:ncRNA | 1 |
| 10 | 413922 | 414285 | + | PF10TR005:ncRNA | 1 |
| 10 | 946361 | 946645 | - | RNAzID:380:ncRNA | 1 |
| 10 | 1239028 | 1239946 | + | PF10TR010:ncRNA | 1 |
| 11 | 392512 | 392586 | - | RNAzID:690:ncRNA | 1 |
| 11 | 1725001 | 1727317 | + | PF11TR009:ncRNA | 1 |
| 11 | 1796491 | 1797637 | + | PF11TR010:ncRNA | 1 |
| 12 | 190102 | 192247 | + | PF12TR001:ncRNA | 1 |
| 12 | 717136 | 717703 | + | PF12TR007:ncRNA | 1 |
| 12 | 1688365 | 1692866 | + | PF12TR013:ncRNA | 1 |
| 12 | 1715852 | 1716018 | - | RNAzID:1301:ncRNA | 1 |
| 13 | 2478559 | 2478698 | + | RNAzID:1833:ncRNA | 1 |
| 13 | 2801236 | 2801355 | - | RNAzID:1895:ncRNA | 1 |
| 14 | 2929611 | 2929760 | + | RNAzID:2385:ncRNA | 1 |
| 14 | 3250638 | 3250955 | + | PF14TR011:ncRNA | 1 |
| 2 | 692518 | 692627 | + | RNAzID:2622:ncRNA | 1 |
| 2 | 933019 | 934006 | + | RNAzID:2720:ncRNA | 1 |
| 3 | 122265 | 127172 | + | PF03TR001:ncRNA | 1 |
| 6 | 1393919 | 1395228 | + | RNAzID:3790:ncRNA | 1 |
| 7 | 101322 | 101843 | + | RNAzID:3887:ncRNA | 1 |
| 7 | 1388551 | 1390315 | + | PF07TR004:ncRNA | 1 |
| 8 | 568310 | 568462 | + | RNAzID:4335:ncRNA | 1 |
| 9 | 1198603 | 1200290 | + | PF09TR005:ncRNA | 1 |
| 9 | 1480862 | 1480935 | + | RNAzID:4815:ncRNA | 1 |

**Supplementary Table 3. Differentially expressed human miRNA detected in exosomes secreted from *P.falciparum*-infected RBCs and uninfected vesicles.**

| **miRNA** | ***Accession #*** | ***p*-value** | **Fold Change [Log_2_]** |
| --- | --- | --- | --- |
| **hsa-miR-98-5p** | MIMAT0000096 | **0.02** | **-2.11** |
| hsa-miR-7-5p | [MIMAT0000252](http://www.mirbase.org/cgi-bin/mature.pl?mature_acc=MIMAT0000252) | 0.04 | -2.21 |
| **hsa-miR-148a-3p** | [MIMAT0000243](http://www.mirbase.org/cgi-bin/mature.pl?mature_acc=MIMAT0000243) | **0.05** | **-2.26** |
| **hsa-let-7g-5p** | [MIMAT0000414](http://www.mirbase.org/cgi-bin/mature.pl?mature_acc=MIMAT0000414) | **0.02** | **-2.28** |
| **hsa-miR-16-5p** | [MIMAT0000069](http://www.mirbase.org/cgi-bin/mature.pl?mature_acc=MIMAT0000069) | **0.04** | **-2.31** |
| **hsa-miR-18b-5p** | [MIMAT0001412](http://www.mirbase.org/cgi-bin/mature.pl?mature_acc=MIMAT0001412) | **0.05** | **-2.38** |
| **hsa-miR-19a-3p** | MIMAT0000073 | **0.03** | **-2.55** |
| **hsa-miR-4732-5p** | [MIMAT0019855](http://www.mirbase.org/cgi-bin/mature.pl?mature_acc=MIMAT0019855) | **0.04** | **-2.58** |
| **hsa-miR-183-5p** | [MIMAT0000261](http://www.mirbase.org/cgi-bin/mature.pl?mature_acc=MIMAT0000261) | **0.01** | **-2.76** |
| hsa-let-7f-5p | [MIMAT0000067](http://www.mirbase.org/cgi-bin/mature.pl?mature_acc=MIMAT0000067) | 0.01 | -2.89 |
| **hsa-let-7a-5p** | [MIMAT0000062](http://www.mirbase.org/cgi-bin/mature.pl?mature_acc=MIMAT0000062) | **0.03** | **-2.94** |
| hsa-miR-210-3p | [MIMAT0000267](http://www.mirbase.org/cgi-bin/mature.pl?mature_acc=MIMAT0000267) | 0.02 | -2.97 |
| **hsa-let-7b-5p** | [MIMAT0000063](http://www.mirbase.org/cgi-bin/mature.pl?mature_acc=MIMAT0000063) | **0.02** | **-2.99** |
| hsa-miR-9-5p | [MIMAT0000441](http://www.mirbase.org/cgi-bin/mature.pl?mature_acc=MIMAT0000441) | 0.04 | -3.07 |
| hsa-miR-367-3p | [MIMAT0000719](http://www.mirbase.org/cgi-bin/mature.pl?mature_acc=MIMAT0000719) | 0.03 | -3.19 |
| hsa-miR-629-5p | [MIMAT0004810](http://www.mirbase.org/cgi-bin/mature.pl?mature_acc=MIMAT0004810) | 0.01 | -3.25 |
| hsa-miR-100-5p | [MIMAT0000098](http://www.mirbase.org/cgi-bin/mature.pl?mature_acc=MIMAT0000098) | 0.02 | -3.35 |
| **hsa-miR-190a-5p** | [MIMAT0000458](http://www.mirbase.org/cgi-bin/mature.pl?mature_acc=MIMAT0000458) | **0.01** | **-3.9** |
| **hsa-miR-218-5p** | [MIMAT0000275](http://www.mirbase.org/cgi-bin/mature.pl?mature_acc=MIMAT0000275) | **0.04** | **-3.99** |
| hsa-miR-125b-5p | [MIMAT0000423](http://www.mirbase.org/cgi-bin/mature.pl?mature_acc=MIMAT0000423) | 0.01 | -4.52 |
| hsa-miR-494-5p | [MIMAT0026607](http://www.mirbase.org/cgi-bin/mature.pl?mature_acc=MIMAT0026607) | 0.04 | -4.67 |
| hsa-miR-323b-5p | [MIMAT0001630](http://www.mirbase.org/cgi-bin/mature.pl?mature_acc=MIMAT0001630) | 0.02 | -5.57 |
| hsa-miR-660-3p | [MIMAT0022711](http://www.mirbase.org/cgi-bin/mature.pl?mature_acc=MIMAT0022711) | 0.03 | -6.06 |
| hsa-miR-532-5p | [MIMAT0002888](http://www.mirbase.org/cgi-bin/mature.pl?mature_acc=MIMAT0002888) | 0.04 | -6.37 |
| hsa-miR-122-5p | [MIMAT0000421](http://www.mirbase.org/cgi-bin/mature.pl?mature_acc=MIMAT0000421) | 0.02 | -7.03 |

*Bold indicates highly abundant miRNA with reads greater than 20 (level of qRT-PCR detection) These results were validated using qRT-PCR.*

**Supplementary Table 4. GO Enrichment analysis: Biological pathways targeted by human miRNA detected to be differentially regulated between exosomes secreted from ring stage P. falciparum-infected RBCs and uninfected vesicles.**

| **Biological regulation functional group** | **Enrichment score** | **Enrichment P value** |
| --- | --- | --- |
| Regulation of cell adhesion | 7.82 | 0.00 |
| Regulation of microtubule-based process | 5 | 0.01 |
| Regulation of establishment or maintenance of cell polarity | 4.85 | 0.01 |
| Regulation of cell cycle | 4.06 | 0.02 |
| Regulation of cell proliferation | 3.52 | 0.03 |

**Supplementary Table 5. gDNA sequencing summary statistics**

| **Exosome Paired-end Sequencing** | |
| --- | --- |
| Total Sequences | 349,577,386 |
| Sequences after removing duplicates | 118,140,710 |
| Sequence length | 100bp |
| % GC | 23 |
| % Mapped to P. falciparum 3D7 | 98.3% |
| % Mapped to human | 0.2% |
| Coverage of Plasmodium falciparum 3D7 reference | 483X |

**Supplementary Table S6:** Taqman microRNA assays purchased from Life Technologies used for the validation of differentially expressed miRNA identified in during small RNA sequencing

| **miRNA** | **Cat#** | **Assay ID** |
| --- | --- | --- |
| hsa-miR-98-5p | 4427975 | [000577](https://www.thermofisher.com/order/genome-database/details/microrna/000577?CID=&ICID=&subtype=microrna_mature) |
| hsa-miR-148a-3p | 4427975 | [000470](https://www.thermofisher.com/order/genome-database/details/microrna/000470?CID=&ICID=&subtype=microrna_mature) |
| hsa-let-7g-5p | 4427975 | 002282 |
| hsa-miR-16-5p | 4427975 | 000391 |
| hsa-miR-18b-5p | 4427975 | 002217 |
| hsa-miR-19a-3p | 4427975 | 000395 |
| hsa-miR-4732-5p | 4427975 | 465097_mat |
| hsa-miR-183-5p | 4427975 | 002269 |
| hsa-let-7a-5p | 4427975 | 000377 |
| hsa-let-7b-5p | 4427975 | 002619 |
| hsa-miR-190a-5p | 4427975 | 000489 |
| hsa-miR-218-5p | 4427975 | 000521 |
| hsa-miR-19b-3p | 4427975 | 000396 |
| hsa-miR-144-3p | 4427975 | 197375_mat |
| hsa-miR-451a | 4427975 | 001141 |
| cel-miR-39 | 4427975 | 000200 |

**Supplementary Figure 11**

Uncorrupted gels and blots:

Figure 3C

Figure 3D

gDNA


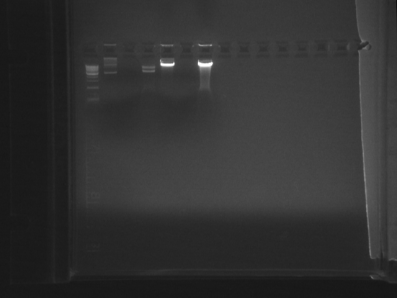


Figure 3D

Ev-DNA


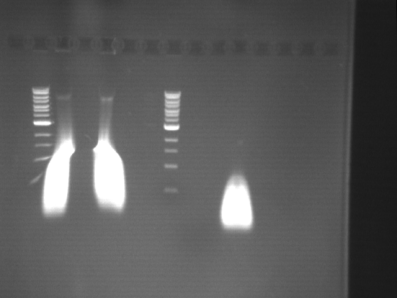


Figure 4A

Top panel


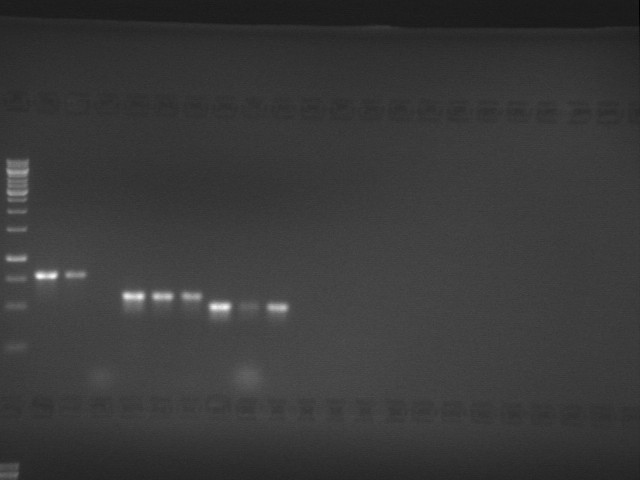


Figure 4A

Bottom panel


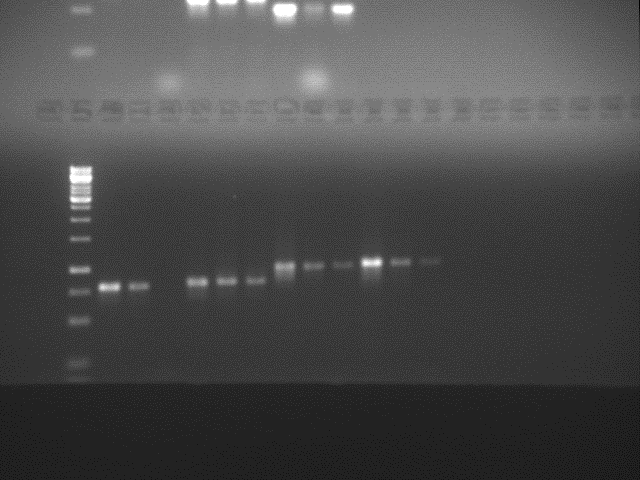


Figure 4D

Anti H4 anti H3


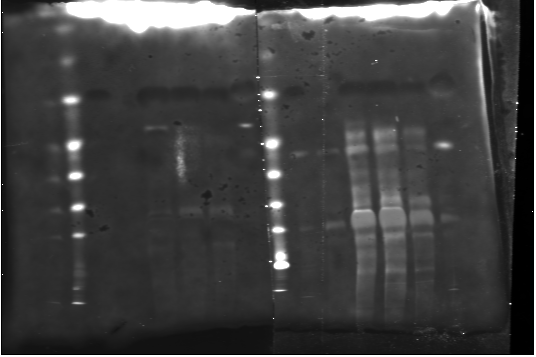


Figure 8C

Anti pTBK1


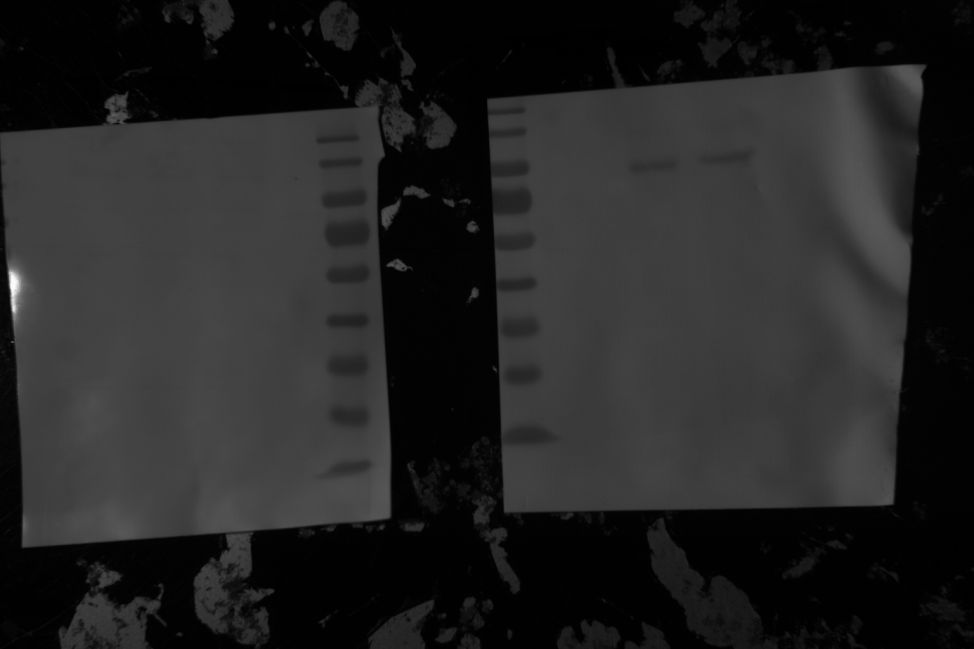


Anti pIRF3


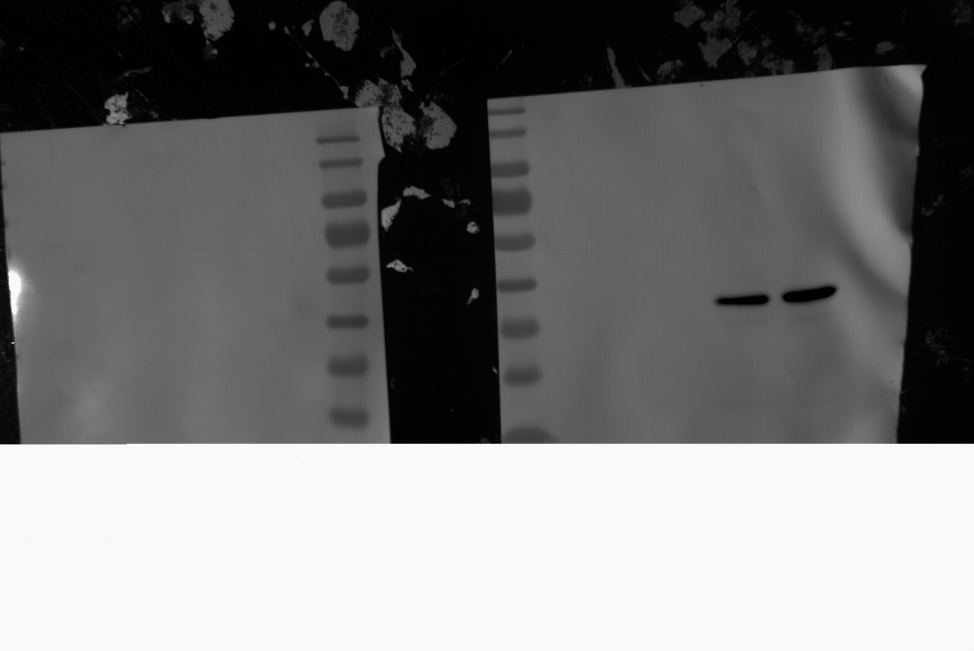


Anti STING


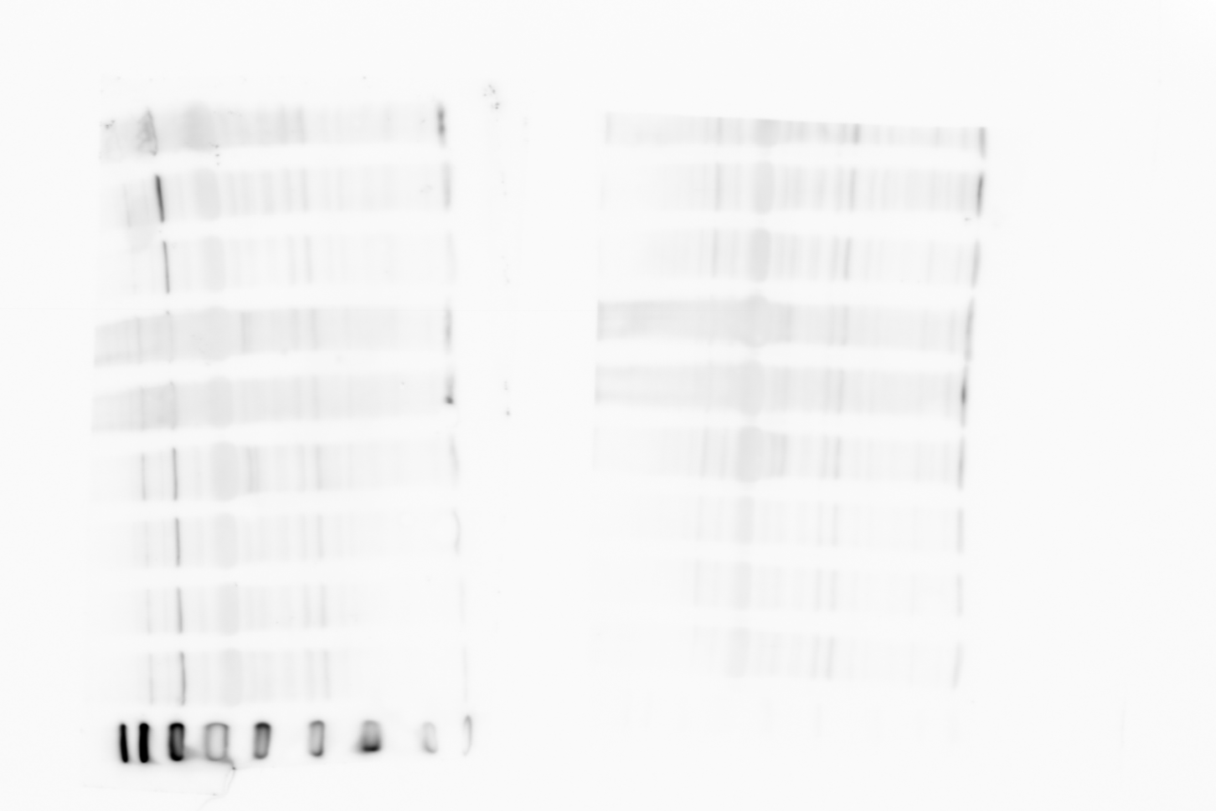


Anti actin


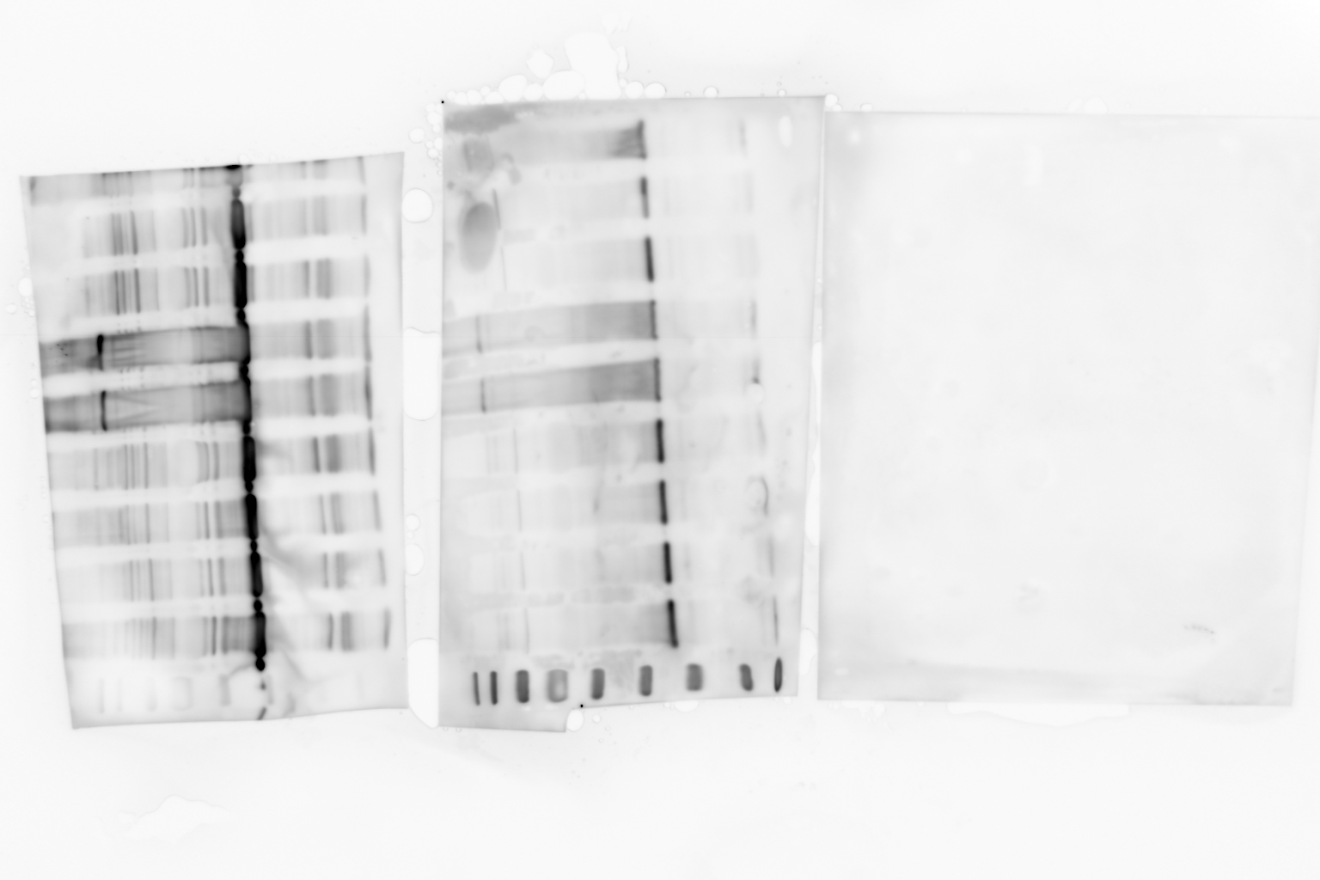


Figure S10

Anti pIRF3


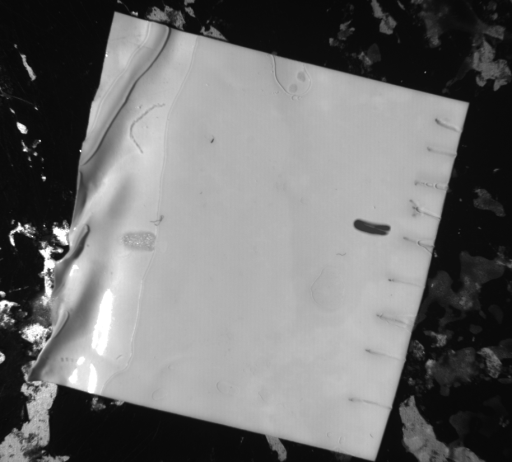


Anti lamin


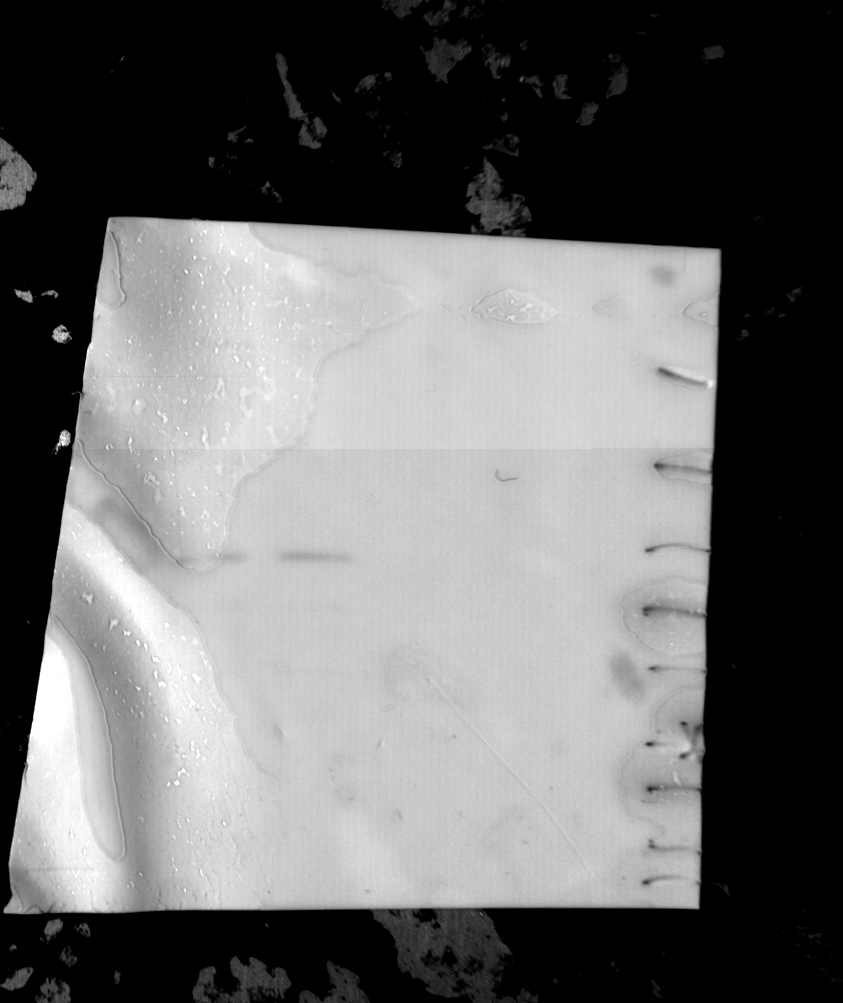


Anti- Tubulin


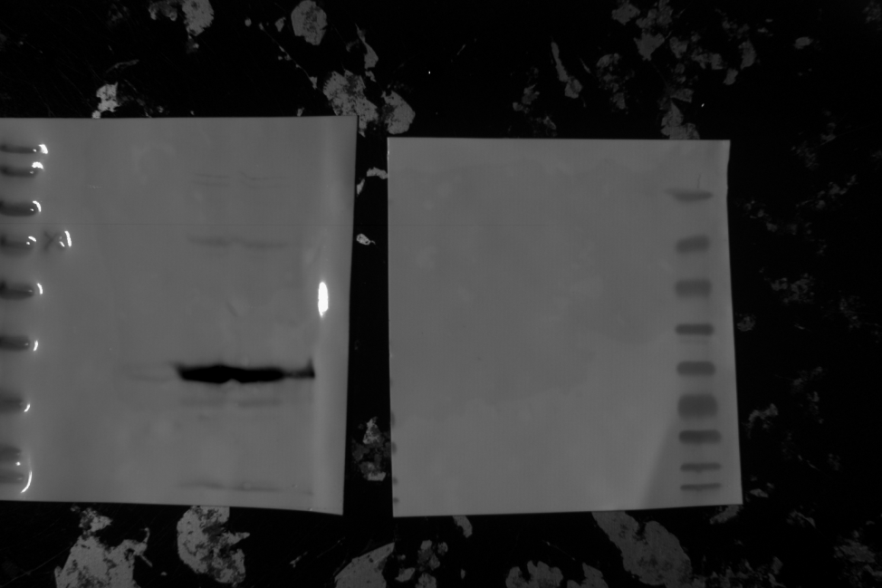

Supplement: Supplementary file 1 — Supplementary information [file 41467_2017_2083_MOESM1_ESM.docx]
